# *FUS* mutations dominate *TBK1* mutations in *FUS*/*TBK1* double-mutant ALS/FTD pedigrees

**DOI:** 10.1007/s10048-021-00671-4

**Published:** 2021-09-13

**Authors:** David Brenner, Kathrin Müller, Serena Lattante, Rüstem Yilmaz, Antje Knehr, Axel Freischmidt, Albert C. Ludolph, Peter M. Andersen, Jochen H. Weishaupt

**Affiliations:** 1 Division of Neurodegeneration, Department of Neurology, Mannheim Center for Translational Neurosciences (MCTN), Medical Faculty Mannheim, Heidelberg University, Theodor-Kutzer-Ufer 1-3, 68167 Mannheim, Germany; 2 Department of Neurology, University of Ulm, Ulm, Germany; 3 Section of Genomic Medicine, Department of Life Sciences and Public Health, Università Cattolica del Sacro Cuore, Rome, Italy; 4 Unit of Medical Genetics, Department of Laboratory and Infectious Disease Sciences, Fondazione Policlinico Universitario A. Gemelli IRCCS, Rome, Italy; 5 Department of Clinical Sciences, Neurosciences, Umeå University, Umeå, Sweden

**Keywords:** Amyotrophic lateral sclerosis, ALS, Frontotemporal dementia, FTD, TBK1, FUS

## Abstract

Mutations in *FUS* and *TBK1* often cause aggressive early-onset amyotrophic lateral sclerosis (ALS) or a late-onset ALS and/or frontotemporal dementia (FTD) phenotype, respectively. Co-occurrence of mutations in two or more Mendelian ALS/FTD genes has been repeatedly reported. However, little is known how two pathogenic ALS/FTD mutations in the same patient interact to shape the final phenotype. We screened 28 ALS patients with a known *FUS* mutation by whole-exome sequencing and targeted evaluation for mutations in other known ALS genes followed by genotype–phenotype correlation analysis of *FUS/TBK1* double-mutant patients. We report on new and summarize previously published *FUS* and *TBK1* double-mutant ALS/FTD patients and their families. We found that, within a family, mutations in *FUS* cause ALS while *TBK1* single mutations are observed in FTD patients. *FUS/TBK1* double mutations manifested as ALS and without a manifest difference regarding age at onset and disease duration when compared to *FUS* single-mutant individuals. In conclusion, *TBK1* and *FUS* variants do not seem to interact in a simple additive way. Rather, the phenotype of *FUS/TBK1* double-mutant patients appears to be dominated by the *FUS* mutation.

**Supplementary Information:**

The online version contains supplementary material available at 10.1007/s10048-021-00671-4.

## Introduction

Amyotrophic lateral sclerosis (ALS) is a devastating motor neuron disease. Its heritability is estimated to be in the range of 30–60%. Overall, mutations in more than 30 genes have been associated with ALS and the clinically and genetically overlapping disease frontotemporal dementia (FTD) in the last decades [1, 2]. Five percent of the patients present with a positive family history for ALS/FTD compatible with an autosomal-dominant and very rarely autosomal-recessive or X-linked dominant Mendelian mode of inheritance. Furthermore, an oligogenic causation in familial and sporadic ALS/FTD is increasingly appreciated [3, 4]. Co-occurrence of mutations in two or more ALS disease genes in the same patient may increase the penetrance of some ALS-associated mutations that have a low effect size. In genetic mouse models, the presence of two different ALS mutations can modify the disease phenotype in a complex manner: We and others have previously shown that expression of mutant *SOD1* or *TARDBP*/TDP-43 in mice interacts with heterozygous loss-of-function (LoF) mutations in *TBK1* to alter the motor neuron disease phenotype in mice [5–7]. However, little is known about the possible interaction of two different ALS mutations in humans. One reason for this is the rarity of identical double mutations in patients, and consequently the small number of patients whose phenotype could be compared to patients with respective single mutations. Moreover, very rarely a pedigree has been published of affected family members carrying mutations in either one or both genes, what would allow phenotypic comparisons on a similar genetic background.

Mutations in the ALS causing genes *FUS* and *TBK1* are observed in about 4% and 2% of familial ALS (FALS) cases in Germany, respectively [8]. Mutations in the RNA-binding protein FUS can cause ALS and FTD in very rare instances. ALS-causing mutations in *FUS* lead to a nucleocytoplasmic redistribution and cytoplasmic aggregation of FUS protein [9, 10]. The heterozygous ALS- and FTD-causing mutations in *TBK1* usually lead to a loss of function of one *TBK1* allele and have been suggested to impair the cellular role of TBK1 in autophagy and glial immune responses [5]. Thus, both genes act most likely at an upstream position in different cellular pathways. It is plausible to hypothesize that the presence of mutations in both genes in the same patient may result in an enhanced penetrance or a synergistic exaggeration of the clinical manifestation. Indeed, we and others have previously described several ALS patients with simultaneous mutations in *FUS* and *TBK1* [11, 12]. In this paper, we compare the co-segregation of genotypes and phenotypes in two families in which mutations in *TBK1* and *FUS* occur separately or in combination and compare them with previously reported and newly identified *TBK1*/*FUS* double-mutant patients.

## Results

### Screening for patients with variants in *FUS* and *TBK1*

Based on previous findings of co-occurrence of *FUS* and *TBK1* mutations in familial ALS patients [11, 12], we screened 28 ALS patients (21 FALS index patients and seven affected relatives) with a known *FUS* mutation by whole-exome sequencing and targeted evaluation for mutations in other known ALS genes. A prior screening for *C9ORF72* had been unsuspicious in these patients. Indeed, we found two additional familial ALS patients with *FUS/TBK1* double mutations: *FUS* c.1540A > G; p.R514G together with *TBK1* c.1328_1331del; p.I443Nfs*3 and *FUS* c.1562G > A; p.R521H in combination with TBK1 c.1522C > A; p.L508I (patients A and C in Table 1). In addition, we found *FUS* mutations to co-occur with missense variants in *ANXA11* (c.772C > T; p.V258M) and *SETX* (c.2113A > C; p.I705L) in two patients (see Supplementary Information and Supplementary Table 1).

**Table 1:** *FUS/TBK1* double-mutant index patients previously described and discovered in this study (AF according to GnomAD)

Pat	ALS type	*FUS* variant		Variation	AF	Evaluation	*TBK1* variant		Variation	AF	Evaluation	Reference
		cDNA	Protein				cDNA	Protein				
A	FALS	c.1540A > G	p.R514G	Missense	0	Pathogenic	c.1328_1331del	p.I443Nfs*3	LoF	0	Pathogenic	This study
B	FALS	c.1570A > G	p.R524G	Missense	0	Pathogenic	c.555 T > A	p.Y185*	LoF	0	Pathogenic	[8, 11]
C	FALS	c.1562G > A	p.R521H	Missense	4E-06	Pathogenic	c.1522C > A	p.L508I	Missense	0.00078	Likely not pathogenic	This study
D	FALS	c.1561C > T	p.R521C	Missense	1E-05	Pathogenic	c.1073G > A	p.R358H	Missense	6.4E-05	Likely not pathogenic	[13]
E	SALS	c.*59G > A	-		4E-06	VUS, but experimental evidence for pathogenicity	c.1445_1446delAT	p.Y482*	LoF	0	VUS	[12]
F	SALS	c.*1998 T > C	-		0.002	VUS	c.2170C > T	p.R724C	Missense	6E-05	VUS, but experimental evidence for pathogenicity	[12]
G	SALS	c.*816delG	-		0	VUS	c.352G > A	p.D118N	Missense	4.2E-06	VUS	[12, 16]

### Overview of previously published *FUS/TBK1* double-mutant families

**Fig. 1:**
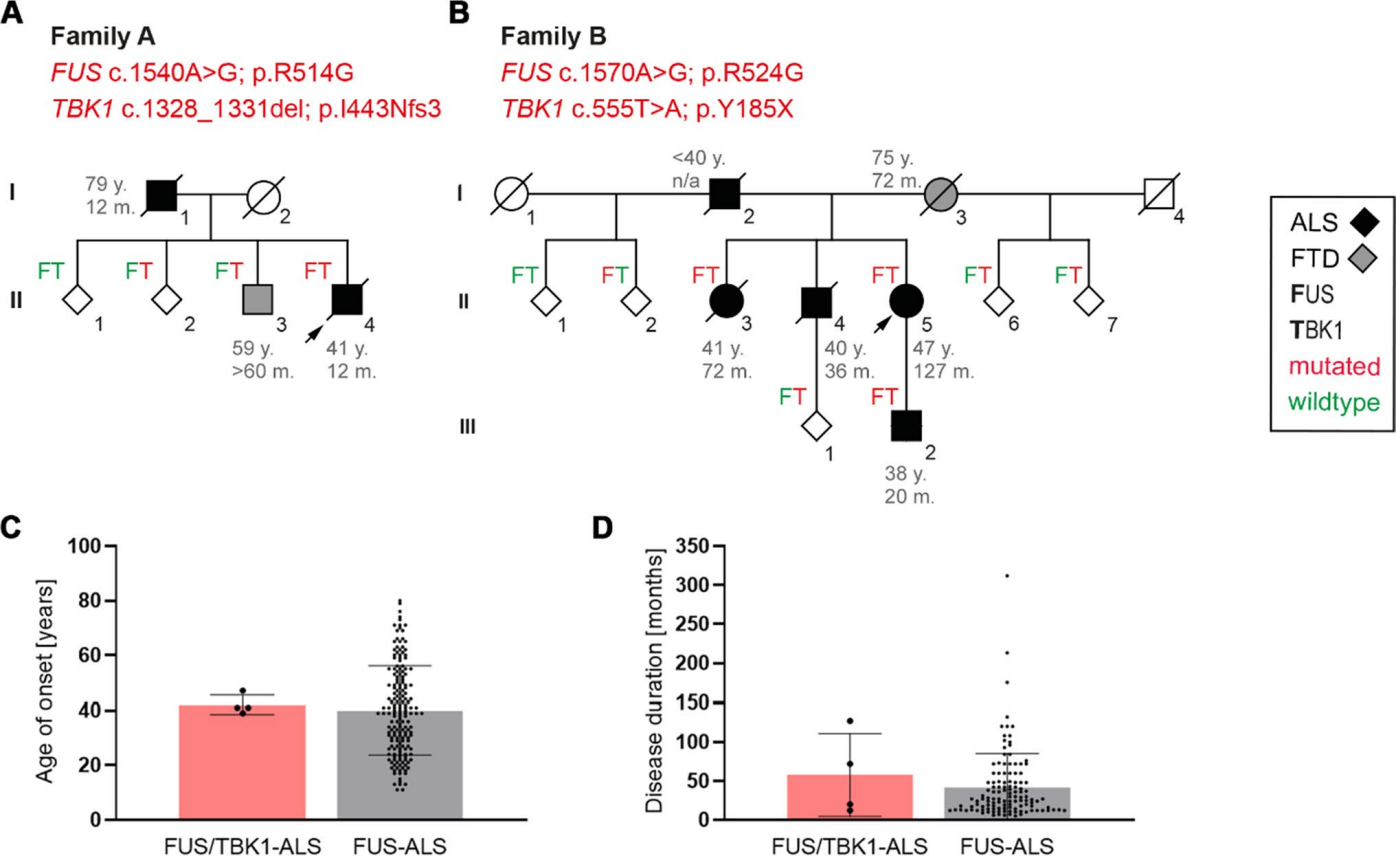
Genotype–phenotype analysis of *FUS/TBK1* double-mutant patients. **A**, **B** Pedigrees of the two FALS families with co-occurrence of mutations in *TBK1* and *FUS*. Arrows indicate index patients. Age at onset (in years) and duration of disease (in months) are indicated next to or below each patient. **C**, **D** Bar graphs showing age of onset and disease duration (time since disease onset until death or tracheostomy) of *FUS/TBK1* double-mutant ALS patients (consisting of patients A II.4, B II.3, B II.5, and B III.2 in Table 2) compared to a large international cohort of FUS-ALS patients

Lattante and colleagues have previously performed genetic testing of 413 Italian ALS patients (32 FALS and 381 SALS) using panel analysis of 32 known ALS/FTD genes [12]. This screen revealed three SALS patients carrying both a *TBK1* variant and a variant in *FUS*. All three *FUS* variants were located in the 3’ UTR in *FUS* (patients E–G in Table 1). Furthermore, de Majo et al. described a *FUS* c.1561C > T; p.R521C together with a *TBK1* c.1073G > A; p.R358H mutation in two first-degree relatives (patient D in Table 1) [13]. In a cohort of 252 whole-exome sequenced German FALS patients, we had previously identified another pedigree with a *TBK1* LoF variant (c.555 T > A; p.Y185*) co-occurring with a *FUS* missense mutation (c.1570A > G; p.R524G) (Fig. 1B; family B in Table 1) [11]. Thus, to our knowledge, at least five index patients have been previously reported who carry both a *TBK1* and a *FUS* variant. Together with the new data from this paper, we summarize different *TBK1* and *FUS* variants found to co-occur in a total of seven index patients, of which four carry a diagnosis of familial ALS. However, the evidence for pathogenicity of the respective mutations differs between these patients. Only in two of the seven *FUS/TBK1* double-mutant index patients (patients A and B in Table 1) pathogenicity can be regarded to be proven for both the *FUS* and the *TBK1* variants (patients A and B), as outlined in detail in Table 1 and the Supplementary Information.

### FUS mutations define the ALS phenotype of *FUS/TBK1* double patients

Our further analysis focuses on the pedigrees with patients with evidence for causality in both *FUS* and *TBK1*, specifically the families of index patients A and B. We performed Sanger sequencing of both genes in available members of the two families and identified two additional *FUS*/*TBK1* double-mutant ALS patients. This resulted in a group of four double-mutant *FUS/TBK1* ALS patients consisting of patients A II.4, B II.3, B II.5, and B III.2 (see Table 2). Compared to an international cohort of 188 FUS-ALS patients [14], this group had a similar mean age at onset of ALS of 42 ± 3.5 (SD) vs. 39.97 ± 16.2 years (*p* = 0.8) and a mean disease duration (time since disease onset until death or tracheostomy) of 57.8 ± 53.3 vs. 41.9 ± 43.6 months (*p* = 0.48) (Fig. 1C and D). However, we point out that the disease characteristics of the cohort in Naumann et al. are prominently influenced by *FUS* mutations that are more aggressive than the mutations p.R514G and p.R524G so that this comparison is admittedly suboptimal. The disease onset in the *FUS/TBK1* double-mutant patients was spinal or bulbar (Table 2). Double-mutant patients did not exhibit comorbid FTD. Although the number of *FUS*/*TBK1* double-mutant patients is low, the present results suggest that a combination of pathogenic variants in *FUS* and *TBK1* does not exacerbate the ALS phenotype compared to pathogenic *FUS* variants only.

**Table 2:** Clinical characteristics of the *FUS/TBK1* double-mutant index patients and available family members

Family/patient			Mutation		Phenotype	Current age	Site of onset	Age of onset	Age of death	Age of IV	Disease duration (m)	Reference
			*FUS*	*TBK1*								
A			c.1540A > G; p.R514G	c.1328_1331del; p.I443N*fs*3								This study
	I.1		n/a	n/a	ALS	†	Bulbar	79	80		12	
	I.2		n/a	n/a	-	†	-	-	-		-	
	II.2			x	-	61	-	-	-		-	
	II.3			x	FTD	64	-	59	-		> 60	
	II.4	(I)	x	x	ALS	†	Bulbar	41	42		12	
B			c.1570A > G; p.R524G	c.555 T > A; p.Y185*								[8, 11]
	I.2		x*		ALS	†	n/a	< 40	40		n/a	
	I.3			x*	FTD	†	-	75	81		72	
	II.2		x		-	72	-	-	-		-	
	II.3		x	x	ALS	†	n/a	41	47		72	
	II.4		n/a	x*	ALS	†	Spinal	40	43		36	
	II.5	(I)	x	x	ALS	60	Spinal	47	-	58	127	
	II.6			x	-	52	-	-	-		-	
	II.7			x	-	56	-	-	-		-	
	III.1			x	-	41	-	-	-		-	
	III.2		x	x	-	39	-	38	-	39	20	
C			c.1562G > A; p.R521H	c.1522C > A; p.L508I	ALS	n/a	n/a	n/a	n/a		n/a	This study
D			c.1561C > T; p.R521C	c.1073G > A; p.R358H	ALS	n/a	n/a	n/a	n/a		n/a	[13]
E			c.*59G > A	c.1445_1446delAT; p.Y482*	ALS	†	Spinal	49	63		168	[12]
F			c.*1998 T > C	c.2170C > T; p.R724C	ALS	†	Bulbar	63	65		24	[12]
G			c.*816delG	c.352G > A; p.D118N	ALS	†	Spinal	81	?		35	[12, 16]

^*^Obligate carrier; (I) Index

^*^IV, invasive ventilation

To gain insight into the phenotypic manifestations of *FUS/TBK1* double variation on a similar genetic background, we compared single- and double-mutant members of families A and B (Table 1). As shown by Fig. 1, the single mutations in *FUS* and *TBK1* co-segregate with ALS or FTD, respectively. In the symptomatic members of both families, the *TBK1* variants alone precipitate a FTD phenotype. By contrast, *FUS* variants alone, or in combination with the *TBK1* variants, cause ALS without FTD, while an obvious additive or synergistic effect of *FUS* and *TBK1* variants on the phenotype is not observed in these two families.

In family A, the likely *FUS* mutant father (I.1) shows an unusually late ALS onset, while his *FUS/TBK1* double-mutant son (II.4) had a very early onset of ALS. In family B, both the obligate *FUS* mutant father (I.2) and the *FUS/TBK1* double-mutant children (II.3 and II.5) and grandson (III.2) show similarly early onsets of ALS (Fig. 1B). Unfortunately, DNA from the family B member II.4 was unavailable. While the disease duration of the ALS patient I.2 with the obligate isolated *FUS* mutation is not known, his children with *FUS/TBK1* double mutations (II.3 and II.5) both displayed a relatively long disease course if compared to the mean survival time of FUS-ALS patients (Fig. 1D). The *FUS/TBK1* double-mutant index patient II.5 of family B has even survived for more than 13 years after ALS onset. By contrast, the grandson (III.2) shows an aggressive disease course.

## Discussion

In this analysis, we extend the previous knowledge about *FUS/TBK1* double-mutant patients by whole-exome sequencing of additional 28 patients with *FUS* mutation. Overall, we report and summarize eight patients with rare variants in both *FUS* and *TBK1*, weigh the evidence for their pathogenicity, and study the phenotype of double-mutant compared to single-mutant ALS patients. Importantly, we report several single- and double-mutant ALS patients in two families, i.e., on a comparable genetic background.

The rationale to perform the extended screening for *TBK1* variants in *FUS* mutation carriers was based on the previous description of patients with concomitant variants in *FUS* and *TBK1* [11, 12]. Our study revealed additional double-mutant individuals, and both *TBK1* and *FUS* belong to the less frequently mutated ALS disease genes. Nevertheless, larger patient numbers will be required to show that mutations in both genes occur more frequently than expected by chance. If not a by chance finding, the penetrance of mutations in *FUS* and *TBK1* could be increased when mutations in both genes co-occur in one individual, thus increasing the probability to be detected in a patient. The 72-year-old asymptomatic *FUS* mutation carrier in family B (II.2) may be regarded as support for a lower penetrance in single-mutant patients.

In our two FUS/ALS families, the combination of *TBK1* and *FUS* variants led to early-onset ALS without FTD comorbidity, while the *TBK1* variants alone caused FTD and the isolated *FUS* variants precipitated early-onset ALS. Possibly, *FUS*/*TBK1* double mutation carriers with early ALS do not survive to the later age at which TBK1-associated FTD usually starts. Consequently, the *FUS* mutations principally shape the disease phenotype in *FUS/TBK1* patients.

A surprising finding of our study is that our initial “double-hit” hypothesis — that the presence of two mutations in the same patient would result in an exacerbated disease through summated neurotoxicity — did not prove true. Comparing the disease characteristics of the group of double *FUS*/*TBK1* mutant ALS patients of this study (four patients) with a large cohort of mostly single *FUS* mutant ALS patients, we found that the age of onset and disease duration were unaltered by the combination of *FUS* and *TBK1* mutations. Considering the apparently complementary pathomechanisms of *FUS* and *TBK1* mutations (in particular impaired protein quality control due to *TBK1* haploinsufficiency and FUS proteinopathy as a consequence of FUS mutations), this is a remarkable finding. Nevertheless, a larger cohort of *FUS/TBK1* double-mutant patients together with mechanistic studies using *FUS/TBK1* double-mutant disease models are warranted to corroborate the findings of our study.

In conclusion, the neurotoxic effects of *TBK1* and *FUS* mutations do not seem to add up in a simple way in patients. Rather, the phenotype of *FUS/TBK1* double-mutant patients appears to be dominated by the *FUS* mutation. These insights may have relevance also for the design of gene-specific therapies for both single- and double-mutant patients.

## Methods

### Patients and ethics statements

All ALS patients were diagnosed according to the EFNS Consensus criteria [15]. The study was approved by the medical ethical review boards of the universities of Ulm and Umea. With informed written consent and in accordance with the Declaration of Helsinki, EDTA blood samples were drawn from controls, ALS patients, and their unaffected relatives during visits at the Neurology departments of the University Hospitals of Ulm and Umea. DNA was extracted from EDTA blood samples according to standard procedures.

### Genotyping of patients for SOD1 and C9ORF72 mutations

Mutations in *SOD1* and *C9ORF72* were excluded prior to exome sequencing of familial ALS cases as described before [11].

### Whole-exome sequencing

Whole-exome sequencing, read mapping, and variant calling were performed on HiSeq2000/2500 systems (Illumina) as described previously [11].

### Statistics

For comparison of two groups, the unpaired two-tailed Student’s *t* test was used. Data are presented as means ± SEM in bar graphs. Statistical significance was reported by the *p* value of the statistical test procedures and was assessed as significant (*, *P* < 0.05), strongly significant (**, *P* < 0.01), or highly significant (***, *P* < 0.001; ****, *P* < 0.0001). All statistical analyses were performed with Prism software (version 9.1.1; GraphPad Software).

## Supplementary Information

Below is the link to the electronic supplementary material.

Supplementary file1 (DOCX 18 KB)

Supplementary file2 (DOCX 16 KB)

## Data Availability

Available upon request.

## References

[1] White MA, Sreedharan J (2016). Amyotrophic lateral sclerosis. Curr Opin Neurol.

[2] Brenner D, Weishaupt JH (2019). Update on amyotrophic lateral sclerosis genetics. Curr Opin Neurol.

[3] Van Blitterswijk M, Van Es MA, Hennekam EAM (2012). Evidence for an oligogenic basis of amyotrophic lateral sclerosis. Hum Mol Genet.

[4] Nguyen HP, Van Broeckhoven C, van der Zee J (2018). ALS genes in the genomic era and their implications for FTD. Trends Genet.

[5] Brenner D, Sieverding K, Bruno C et al (2019) Heterozygous Tbk1 loss has opposing effects in early and late stages of ALS in mice. J Exp Med jem.20180729. 10.1084/jem.20180729 10.1084/jem.20180729 PMC6363427 30635357

[6] Gerbino V, Kaunga E, Ye J (2020). The loss of TBK1 kinase activity in motor neurons or in all cell types differentially impacts ALS disease progression in SOD1 mice. Neuron.

[7] Sieverding K, Ulmer J, Bruno C et al (2021) Hemizygous deletion of Tbk1 worsens neuromuscular junction pathology in TDP-43G298S transgenic mice. Exp Neurol 335. 10.1016/j.expneurol.2020.113496 10.1016/j.expneurol.2020.113496 33038415

[8] Müller K, Brenner D, Weydt P et al (2018) Comprehensive analysis of the mutation spectrum in 301 German ALS families. J Neurol Neurosurg Psychiatry jnnp-2017-317611. 10.1136/jnnp-2017-317611 10.1136/jnnp-2017-317611 29650794

[9] Kwiatkowski TJ, Bosco DA, LeClerc AL (2009). Mutations in the FUS/TLS gene on chromosome 16 cause familial amyotrophic lateral sclerosis. Science (80- ).

[10] Vance C, Rogelj B, Hortobágyi T (2009). Mutations in FUS, an RNA processing protein, cause familial amyotrophic lateral sclerosis type 6. Science (80- ).

[11] Freischmidt A, Wieland T, Richter B (2015). Haploinsufficiency of TBK1 causes familial ALS and fronto-temporal dementia. Nat Neurosci.

[12] Lattante S, Doronzio PN, Marangi G (2019). Coexistence of variants in TBK1 and in other ALS-related genes elucidates an oligogenic model of pathogenesis in sporadic ALS. Neurobiol Aging.

[13] de Majo M, Topp SD, Smith BN (2018). ALS-associated missense and nonsense TBK1 mutations can both cause loss of kinase function. Neurobiol Aging.

[14] Naumann M, Peikert K, Günther R (2019). Phenotypes and malignancy risk of different FUS mutations in genetic amyotrophic lateral sclerosis. Ann Clin Transl Neurol.

[15] Andersen PM, Abrahams S, Borasio GD (2012). EFNS guidelines on the clinical management of amyotrophic lateral sclerosis (MALS)—revised report of an EFNS task force. Eur J Neurol.

[16] Pozzi L, Valenza F, Mosca L (2017). TBK1 mutations in Italian patients with amyotrophic lateral sclerosis: genetic and functional characterization. J Neurol Neurosurg Psychiatry.

