# Supplementary material for: FUS mutations dominate TBK1 mutations in FUS/TBK1 double-mutant ALS/FTD pedigrees
Source: Neurogenetics. 2021 Sep 13;23(1):59–65. doi: 10.1007/s10048-021-00671-4 (PMC8782814; doi:10.1007/s10048-021-00671-4)
Supplement: Supplementary file 1 — Supplementary file1 (DOCX 18 KB) [file 10048_2021_671_MOESM1_ESM.docx]

**Supplementary information**

***FUS* mutations dominate *TBK1* mutations in *FUS*/*TBK1* double mutant ALS/FTD pedigrees**

Brenner et al.

**Combination of *FUS* mutations with mutations in ALS genes other than *TBK1***

We found the combination of *FUS* c.1483C>T; p.R495* with *ANXA11* c.772C>T; p.V258M in one patient and *FUS* c.1562G>A; p.R521H with *SETX* c.2113A>C; p.I705L in two affected siblings. Both *FUS* variants have repeatedly been described in ALS patients and are to be regarded as pathogenic [1]. The missense variant in *ANXA11* is very rare (does not occur in the GnomAD dataset) and has to be classified as a variant of unknown significance (VUS), while the comparatively frequent missense variant in *SETX* is predicted as benign (Suppl. Table 1).

**Evaluation of pathogenicity of *FUS* and *TBK1* variants observed in combination**

FUS variants:

The *FUS* variant c.1570A>G; p.R524G is in a mutational hot spot in the nuclear localization sequence of *FUS* [2]. It is absent in control cohorts and co-segregates with disease in three affected members of this family (Fig. 1 B), supporting a pathogenic role [2]. *FUS* variant c.1561C>T; p.R521C has previously been observed in several ALS patients with a particularly aggressive disease course and is classified as pathogenic [3, 4]. Finally, the *FUS* variants c.1540A>G; p.R514G and c.1562G>A; p.R521H have been demonstrated to co-segregate with ALS [4, 5]. Considering the lack of co-segregation data or statistically significant enrichment in patients, the three different *FUS* 3’ UTR variants c.*59G>A, c.*1998T>C, and c.*816delG in sporadic patients (patients E-G in Table 1) have to be formally classified as variants of unknown significance (VUS). However, the variant c.*59G>A has been shown to result in strong FUS overexpression and nucleocytoplasmic translocation in patient cells [6], lending solid support for a pathogenic role.

TBK1 variants:

While nonsense loss-of-function mutations (patients A, B, E in Table 1) in *TBK1* can be considered pathogenic for ALS [7], evidence for causality of missense variants is less clear. The *TBK1* c.1073G>A; p.R358H variant occurred in two first-degree relatives, but strong evidence for causality of this missense variant is lacking [8]. The *TBK1* missense variant c.2170C>T; p.R724C is located in a functionally critical protein domain that is necessary for binding of TBK1 to autophagy adaptor proteins, such as optineurin, although lack of optineurin binding of TBK1 p.R724C has not been experimentally proven. Nevertheless, considering haploinsufficiency of *TBK1* together with the fact that this variant reduced the TBK1 protein expression significantly to about 60% [9], an at least partial contribution to ALS causation in a bi- or oligogenic mode is likely. The *TBK1* variant c.1522C>A; p.L508I is in the GnomAD database at a minor allele frequency (MAF) of 0.08%. Since evidence for causality is lacking, it is classified as VUS. The *TBK1* variant c.352G>A; p.D118N located in the kinase domain is rare (MAF 0.0004 %) and has been shown to reduce TBK1 phosphorylation activity without affecting protein stability [10]. However, as it is unproven whether the kinase activity of TBK1 is the ALS-relevant function, and due to lack of co-segregation data, it also is classified as a VUS.

**References**

1. Naumann M, Peikert K, Günther R, et al (2019) Phenotypes and malignancy risk of different FUS mutations in genetic amyotrophic lateral sclerosis. Ann Clin Transl Neurol 6:2384–2394. https://doi.org/10.1002/acn3.50930
2. Müller K, Brenner D, Weydt P, et al (2018) Comprehensive analysis of the mutation spectrum in 301 German ALS families. J Neurol Neurosurg Psychiatry jnnp-2017-317611. https://doi.org/10.1136/jnnp-2017-317611
3. Kwiatkowski TJ, Bosco DA, LeClerc AL, et al (2009) Mutations in the FUS/TLS gene on chromosome 16 cause familial amyotrophic lateral sclerosis. Science (80- ) 323:1205–1208. https://doi.org/10.1126/science.1166066
4. Vance C, Rogelj B, Hortobágyi T, et al (2009) Mutations in FUS, an RNA processing protein, cause familial amyotrophic lateral sclerosis type 6. Science (80- ) 323:1208–1211. https://doi.org/10.1126/science.1165942
5. Waibel S, Neumann M, Rosenbohm A, et al (2013) Truncating mutations in FUS/TLS give rise to a more aggressive ALS-phenotype than missense mutations: A clinico-genetic study in Germany. Eur J Neurol 20:540–546. https://doi.org/10.1111/ene.12031
6. Sabatelli M, Moncada A, Contel A, et al (2013) Mutations in the 3′ untranslated region of FUS causing FUS overexpression are associated with amyotrophic lateral sclerosis. Hum Mol Genet 22:4748–4755. https://doi.org/10.1093/hmg/ddt328
7. Freischmidt A, Müller K, Ludolph AC, et al (2017) Association of Mutations in TBK1 With Sporadic and Familial Amyotrophic Lateral Sclerosis and Frontotemporal Dementia. JAMA Neurol 74:110. https://doi.org/10.1001/jamaneurol.2016.3712
8. de Majo M, Topp SD, Smith BN, et al (2018) ALS-associated missense and nonsense TBK1 mutations can both cause loss of kinase function. Neurobiol Aging 71:266.e1-266.e10. https://doi.org/10.1016/j.neurobiolaging.2018.06.015
9. Lattante S, Doronzio PN, Marangi G, et al (2019) Coexistence of variants in TBK1 and in other ALS-related genes elucidates an oligogenic model of pathogenesis in sporadic ALS. Neurobiol Aging 84:239.e9-239.e14. https://doi.org/10.1016/j.neurobiolaging.2019.03.010
10. Pozzi L, Valenza F, Mosca L, et al (2017) TBK1 mutations in Italian patients with amyotrophic lateral sclerosis: Genetic and functional characterization. J Neurol Neurosurg Psychiatry 88:869–875. https://doi.org/10.1136/jnnp-2017-316174
