# Supplementary material for: FUS mutations dominate TBK1 mutations in FUS/TBK1 double-mutant ALS/FTD pedigrees
Source: Neurogenetics. 2021 Sep 13;23(1):59–65. doi: 10.1007/s10048-021-00671-4 (PMC8782814; doi:10.1007/s10048-021-00671-4)
Supplement: Supplementary file 2 — Supplementary file2 (DOCX 16 KB) [file 10048_2021_671_MOESM2_ESM.docx]

| **Suppl. Table 1**: FUS mutant patients with co-occurrent variant in another ALS gene (other than *TBK1*). | | | | | | | | | | |  |  |  |
| --- | --- | --- | --- | --- | --- | --- | --- | --- | --- | --- | --- | --- | --- |
|  |  |  |  |  |  |  |  |  |  |  |  |  |  |
| ***Pat.*** | ***ALS type*** | ***FUS variant*** | | ***Variation*** | ***AF*** | ***Evaluation*** | ***Other gene variant*** | | | ***Variation*** | ***AF*** | ***Evaluation*** | ***Reference*** |
|  |  | ***cDNA*** | ***Protein*** |  |  |  | ***Gene*** | ***cDNA*** | ***Protein*** |  |  |  |  |
| **1** | FALS | c.1483C>T | p.R495* | LoF | 8,1E-06 | pathogenic | ANXA11 | c.772C>T | p.V258M | missense | 0 | VUS | [2] |
| **2** | FALS | c.1562G>A | p.R521H | missense | 4E-06 | pathogenic | SETX | c.2113A>C | p.I705L | missense | 0 | benign | [2] |
